# Eliminating the Attentional Blink through Binaural Beats: A Case for Tailored Cognitive Enhancement

**DOI:** 10.3389/fpsyt.2015.00082

**Published:** 2015-06-04

**Authors:** Susan A. Reedijk, Anne Bolders, Lorenza S. Colzato, Bernhard Hommel

**Affiliations:** ^1^Institute for Psychological Research, Leiden Institute for Brain and Cognition, Leiden University, Leiden, Netherlands

**Keywords:** attentional blink, dopamine, attention, binaural beats, neurotransmitters, cognitive enhancement

## Abstract

Enhancing human cognitive performance is a topic that continues to spark scientific interest. Studies into cognitive-enhancement techniques often fail to take inter-individual differences into account, however, which leads to underestimation of the effectiveness of these techniques. The current study investigated the effect of binaural beats, a cognitive-enhancement technique, on attentional control in an attentional blink (AB) task. As predicted from a neurocognitive approach to cognitive control, high-frequency binaural beats eliminated the AB, but only in individuals with low spontaneous eye-blink rates (indicating low striatal dopamine levels). This suggests that the way in which cognitive-enhancement techniques, such as binaural beats, affect cognitive performance depends on inter-individual differences.

## Introduction

For ages, humans have used cognitive and physical interventions to improve their performance, and new cognitive-enhancement techniques are announced daily. Unfortunately, most of them do not stand scientific evaluation (1), with the recent failure to find an effect of brain training in more than 11,000 participants (2) being a particularly attention-grabbing example. And yet, many tests are likely to systematically underestimate the potential of enhancement techniques by ignoring inter-individual differences in cognitive or neural parameters that determine or reflect individual sensitivity to interventions.

One indication that individual differences play an important role in the degree to which enhancement techniques affect cognition is the observation that the widely assumed creativity-enhancing effect of positive mood (3) is mediated by individual differences related to dopamine levels. As demonstrated by Akbari Chermahini and Hommel (4), individuals with low dopamine levels (as assessed by spontaneous eye-blink rates; EBRs) show better performance in a divergent-thinking task after the induction of positive mood, while individuals with medium or high dopamine levels show no effect. Accordingly, studies that neglect functionally relevant individual differences can be expected to replicate or fail to replicate the assumed connection between creativity and mood, depending on the specific characteristics of the given sample – which explains the great inconsistency between the available studies [see Ref. (3)].

The present study assessed an enhancement technique that has been claimed to target cognitive-control functions: binaural beats – the subjective experience of a beating tone with a frequency that corresponds to the frequency difference between two binaurally presented tones (5). As first reported by Heinrich Wilhelm Dove in 1839, presenting two sounds of slightly different frequencies (330 and 350 Hz, say) to the left and right ear results in the integrated perception of one sound that fluctuates in a frequency that corresponds to the difference (20 Hz). While the neural mechanisms underlying this illusion remain unknown, a role of the reticular activation system has been proposed (6). In animals, binaural beat-inducing conditions are associated with spreading neural patterns of phase locking in the auditory cortex, which ultimately result in specific firing patterns in the inferior colliculus that could reflect a binaural beat (7, 8).

Even though weaker than the neural response to beats physically present in the signal (9), binaural beats elicit a similar neural response as real acoustic beats (7, 8, 10, 11). This suggests that the illusion of a beating tone arises through neural pathways normally associated with binaural sound detection in the environment (7, 9). In humans, the neural phase locking that binaural beats elicit can influence ongoing cognitive processing. Low-frequency binaural beats are associated with mental relaxation and high-frequency beats with alertness and attentional concentration (1, 6). This suggests that high-frequency beats might facilitate attentional control, which would fit with the observation that high-frequency neurofeedback training over the frontal cortex improves attentional efficiency (12).

We tested this hypothesis by using the attentional blink (AB) task, in which two targets (T1 and T2) are presented in a rapid stimulus stream. While participants are commonly able to report T1, they often miss T2 if it appears soon after T1 [(13); for a review, see Ref. (14)]. While factors such as T1 target identification, consolidation, and response selection also play a role (15), the AB mainly has been attributed to the suboptimal allocation of attentional resources to the two targets: too much emphasis on T1 leaves too few resources for T2 (16–19). Indeed, interventions that supposedly improve attentional control, like meditational practice, have been shown to reduce the AB (20).

Cognitive-control functions, such as those needed in the AB task, rely on the interplay between two dopaminergic pathways: a frontal pathway driving working memory and top-down control functions, and a striatal pathway supporting mental flexibility and bottom-up interruptions (21). Interestingly for our purposes, people can be biased toward one or the other pathway. Those who are biased toward the frontal dopamine pathway show high prefrontal but low striatal dopamine levels, while those biased toward the striatal pathway show evidence of low prefrontal but high striatal dopamine levels [for reviews, see Ref. (22, 23)] – with the latter being associated with higher spontaneous eye-blink rates [EBRs; see Ref. (24, 25)]. Also of interest, individual differences in the balance between these two pathways in the corresponding functions have been demonstrated to modulate the size of the AB: it is smaller in individuals with high working-memory span (26) – a frontal function (21) – and with a genetic setup that is associated with high frontal and low striatal dopamine levels (27).

In the present study, we used EBR, a well-established clinical indicator (28) thought to index dopamine (DA) production in the striatum (29–31), to assess individual (striatal) dopaminergic functioning. The idea that EBR mirrors dopaminergic functioning is first of all supported by clinical observations in patients with DA-related dysfunctions. For example, EBRs are elevated in schizophrenia patients (32), who in PET studies showed elevated striatal dopamine uptake, both on and off medication (33, 34). By contrast, EBRs are reduced in recreational cocaine users (35), and in Parkinson’s patients (36) – two populations suffering from reduced functioning of D2 receptors and severe losses of nigrostratial dopaminergic cells, respectively (37, 38). Further, Colzato et al. (39) showed that the level of Psychoticism, which has been associated with dopaminergic activity (40), was predicted by EBR: people with higher scores on the Psychoticism scale showed higher EBRs. Second, pharmacological studies in non-human primates and humans have shown that DA agonists, as apomorphine, and antagonists increase and decrease EBRs, respectively (29, 41). Third, a genetic study in humans demonstrated a strong association between EBR and the DRD4/7 genotype, which is related to the control of striatal DA release (24). Above all, a recent study employing PET (42) has shown that baseline EBR was positively related to striatal D2-like receptor availability throughout the striatum. Accordingly, we assumed EBR to indicate an individual bias toward the frontal (low EBR) or the striatal pathway (high EBR).

How might individual differences related to the striatal dopamine level modulate enhancement effects? Given the evidence that high frontal and low striatal dopamine levels are associated with better performance the AB task (27), enhancing cognitive control would be expected to induce or increase the bias toward the frontal pathway. It might seem obvious to assume that the benefits are strongest for those who need them most, that is, for individuals with a bias toward the striatal pathway (i.e., with higher EBRs). As these individuals are more drawn to cognitive flexibility than to cognitive control, a stimulus that enhances cognitive control might affect them more than individuals who already favor control over flexibility. However, recent enhancement studies with genetic predictors suggest that more reliable enhancement can be found in individuals with a more suitable predisposition [i.e., are, genetically or otherwise, predisposed toward the cognitive process(es) the present task calls for; (43)], suggesting that more enhancement might be found in individuals with a stronger bias toward the frontal cognitive-control pathway (i.e., individuals with lower EBRs). We tested these possibilities by comparing AB performance in individuals with low and high EBRs, during exposure to binaural beats with low (alpha) or high (gamma) frequency, or a constant tone as control.

## Materials and Methods

Twenty-four students (22 female, 2 male; aged 17–25 years old) of Leiden University participated in this study in exchange for course credit or pay. All had normal or corrected-to-normal sight and hearing. Participants were considered suitable to participate in this study if they fulfilled the following criteria: (i) age between 17 and 30 years; (ii) no history of neurological or psychiatric disorders; (iii) no history of substance abuse or dependence. After the study was explained to them by the experimenter, written informed consent was obtained from all participants. In the case of one underage participant, written informed consent was also obtained from their parents/caretakers. The study was approved by the Leiden University Ethics Committee of the Institute of Psychology.

As mood correlates with dopamine levels (4), we controlled for mood by means of a 9 × 9 arousal/pleasure affect grid (44). Participants took part in three sessions in counterbalanced order, where they listened to alpha-frequency (10 Hz) or gamma-frequency (40 Hz) binaural beats, or a constant tone of 340 Hz (control condition), all embedded in white noise to enhance clarity of the beats (5), for 3 min before and during the AB task. Binaural beats were presented through in-ear headphones (Etymotic Research ER-4B microPro), which provide 35 dB noise attenuation. Both binaural beat conditions were based on a 340 Hz carrier tone, which was used as the constant tone in the control condition. EBRs were measured at the beginning of each session for 5 min before presentation of the binaural beats, using six Ag/AgCl electrodes: two placed next to each eye (measuring saccades), and two placed above and below the right eye (measuring the blink). Two electrodes placed on the mastoids served as a reference. Participants were instructed to relax and look (but not stare!) straight ahead at a paper with a fixation cross that was taped to the computer monitor. This monitor was turned off during the EBR measurement. As EBR is stable during the day but goes up in the evening, participants were never tested after 7 p.m. (45). Before analysis, the three EBR measurements were averaged to create one stable EBR count for every participant.

In the AB task, two digit targets (drawn from 2 to 9) and 18 letter distracters (randomly drawn from A to Z) were presented for 70 ms each with an inter-stimulus interval of 30 ms at the center of a 19-inch CRT monitor (1024 × 768 pixels, 100 Hz vertical refresh rate). To reduce predictability, the position of T1 in the rapid serial visual presentation (RSVP) stream varied randomly between positions 7, 8, and 9. Participants were to report T1 before T2 by pressing the corresponding number keys on a keyboard. T2 appeared for 100, 300, 500, and 800 ms after T1 (lags 1, 3, 5, and 8, respectively).

## Results

As a repeated measures analysis of variance (ANOVA) did neither find any differences in pre-experimental EBR between sessions, *F*(2, 46) = 1.23, *p* = 0.303, ${\text{η}}_{\text{p}}^{\text{2}}=0.05$, nor between binaural beat conditions, *F*(2, 46) = 1.77, *p* = 0.18, ${\text{η}}_{\text{p}}^{\text{2}}=0.07$, two groups of 12 participants each were created by median (17.4) split of the average EBR score over all three sessions. The groups’ mood did not differ in both arousal or pleasure, *F*s(2, 22) < 1. A mixed ANOVA (Greenhouse–Geisser corrected where necessary) of conditional T2 performance (T2|T1) yielded a three-way interaction between EBR group, stimulation, and lag, *F*(3.04, 66.95) = 3.38, *p* = 0.023, ${\text{η}}_{\text{p}}^{\text{2}}=0.13$. Separate analyses revealed lag effects in the low-EBR group, *F*(1.28, 14.03) = 9.35, *p* = 0.006, ${\text{η}}_{\text{p}}^{\text{2}}=0.46$, and the high-EBR group, *F*(1.35, 14.85) = 10.33, *p* = 0.003, ${\text{η}}_{\text{p}}^{\text{2}}=0.48$, reflecting the standard AB pattern. This effect interacted with stimulation in the low-EBR group, *F*(2.52, 27.66) = 3.59, *p* = 0.032, ${\text{η}}_{\text{p}}^{\text{2}}=0.25$, but not in the high-EBR group, *F*(2.73, 29.99) < 1.

**Figure 1:**
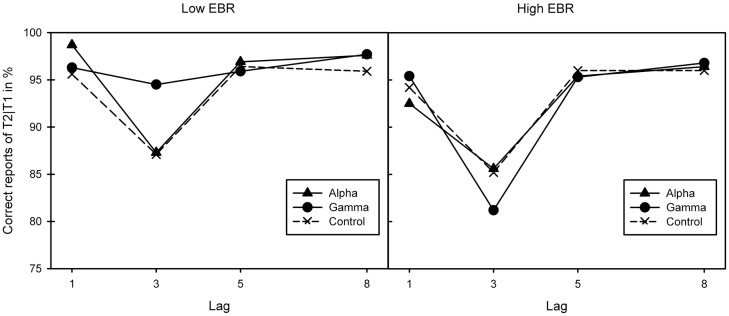
**Correct report of T2|T1 for the three binaural beat conditions (alpha, gamma, and control) in the two EBR groups**. Lag effects in both the low [left-hand graph; *F*(1.28, 14.03) = 9.35, *p* = 0.006, ${\text{η}}_{\text{p}}^{\text{2}}=0.46$] and high-EBR groups [right-hand graph; *F*(1.35, 14.85) = 10.33, *p* = 0.003, ${\text{η}}_{\text{p}}^{\text{2}}=0.48$] reflective of the standard attentional blink pattern. This interacted with stimulation in the low-EBR group [*F*(2.52, 27.66) = 3.59, *p* = 0.032, ${\text{η}}_{\text{p}}^{\text{2}}=0.25$] but not in the high-EBR group [*F*(2.73, 29.99) < 1].

**Figure 2:**
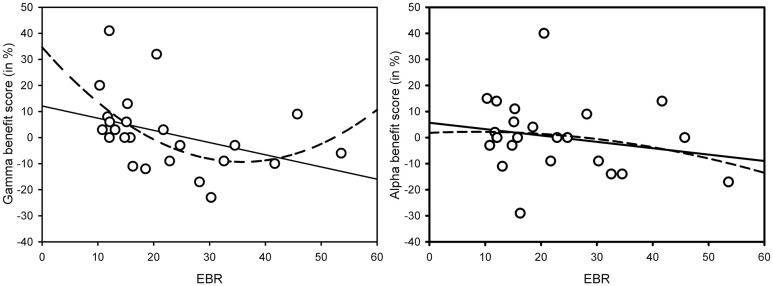
**Linear (solid line) and quadratic (dotted line) relationships between gamma-frequency AB benefit scores on lag 3 and EBR [left-hand graph – linear: *F*(1, 22) = 3.89, *p* = 0.061; quadratic: *F*(2, 21) = 3.84, *p* = 0.038] and alpha-frequency AB benefit scores on lag 3 and EBR [right-hand graph – linear: *F*(1, 22) = 1.03, *p* = 0.32; quadratic: *F*(2, 21) < 1]**. Benefit scores were calculated by subtracting lag 3 AB performance in the control condition from lag 3 AB performance in the (gamma or alpha) binaural beat condition.

As Figure 1 suggests, the interaction in the low-EBR group was produced by the gamma condition (and disappeared in an ANOVA without that condition, *p* > 0.58), where the AB was no longer reliable, *p* > 0.1. To get a better view of the relationship between EBR and gamma-frequency stimulation, we calculated a gamma benefit variable by subtracting lag 3 accuracy in the control condition from lag 3 accuracy in the gamma condition, and entered these variables in a regression analysis. Given that cognitive performance has been shown to relate to EBR in non-linear ways [leading to quadratic relationships: (46)], we computed both linear and quadratic fits. As shown in Figure 2, there exists a positive quadratic relationship between EBR and benefit from gamma stimulation on lag 3, *F*(2, 21) = 3.84, *p* = 0.038, while the linear relationship failed to reach significance, *F*(1, 22) = 3.89, *p* = 0.061. The same analysis was done with an alpha benefit variable, but neither the linear, *F*(1, 22) = 1.03, *p* = 0.32, nor the quadratic relationship, *F*(2, 21) < 1, reached significance^1^.

## Conclusion

As expected, we found evidence for the enhancement of attentional control through high-frequency binaural beats. Given that the AB is considered a very robust effect, it is remarkable that gamma beats eliminated this effect altogether. Importantly, the success of the enhancement intervention was predicted by EBR, our marker of the individual striatal dopamine level. While no enhancement was observed for people with high EBRs (which we take to indicate a bias toward the striatal pathway), reliable enhancement was obtained in individuals with low EBRs (presumably indicating a bias toward the frontal pathway). This suggests that enhancement techniques like binaural beats are unable to compensate for unfavorable control styles or dispositions but rather further improve individuals that have a suitable control style or disposition in place already – a conclusion that fits with the implications of a recent training study (43).

A possible explanation for why gamma-frequency entrainment eliminates the AB in individuals with a low EBR could be that presenting these individuals with gamma binaural beats leads to temporarily increased activity in the gamma band during presentation of T1 and T2, inside and outside of the AB period. Normally, increased gamma synchronization on the EEG only occurs after presentation of T1 and after presentation of T2 outside of the AB period, while this gamma peak does not occur after presentation of T2 inside the AB period [300 ms after presentation of T1; (47)]. This suggests that, at least in people with an already favorable cognitive control style, gamma-frequency binaural beats enhance gamma synchronization in neural firing, not only on non-AB trials but also during the sensitive AB period.

Another possible explanation is that the gamma beat successfully distracts individuals who otherwise would allocate too many resources to T1 (16–19). If individuals with a low EBR (who most likely favor the frontal dopamine pathway) are more sensitive to the distracting gamma-frequency beat, they would indeed show a larger decrease in AB than individuals with high EBRs (who likely favor the striatal dopamine pathway). However, given that we did not use EEG equipment to measure the brain’s physiological responses to the binaural beats, both these explanations remain speculative for now.

The finding that gamma-frequency binaural beats attenuate the AB in individuals with a low EBR is itself somewhat at odds with previous literature. Gamma frequency on the EEG is typically associated with greater attentional investment (12, 48), which in turn is associated with a deeper blink (16). However, as the blink most likely arises from suboptimal allocation of resources over T1 and T2 (18, 19), it seems to be that external gamma-frequency stimulation can for some individuals, depending on their striatal dopamine level, aid in proper allocation of resources (20). Another possible explanation is that cognitive-enhancement methods that target gamma-band activity in the brain can successfully overstimulate and thereby distract some individuals from the task, which reduces the blink (16).

Why alpha-frequency binaural beats did not affect AB performance for neither the low- nor the high-EBR group remains unclear for now. It is possible, however, that all participants experienced a form of alpha entrainment in all conditions: in the AB task, items in the RSVP stream are typically presented with a frequency of 10 items per second, which equals visual entrainment at alpha frequency (49). Therefore, even participants in the control condition experienced some form of alpha entrainment. While it is unclear whether visual and auditory entrainment methods exert comparable effects on the brain (1), it cannot be ruled out that the auditory entrainment at alpha level that we presented in this study was redundant: participants already experienced alpha entrainment from the visual stimuli.

One limitation of our study is that our sample was imbalanced with respect to gender. While the available evidence does not suggest that this might have had a systematic impact on the findings, it seems to be important to replicate our results in a sample of male participants. Another limitation relates to the fact that our division of the sample in two groups based on spontaneous EBR is artificial: EBR is a continuous construct with no clear line between high and low values (24, 30). This division into two groups has caused its participants to share more characteristics than just EBR. For instance, sleep-deprived individuals show higher EBRs (50), so it is possible that the high-EBR group featured more individuals with sleeping problems than the low-EBR group. Also, while high EBRs seem to be related to schizophrenia (30), there are individuals showing abnormally high EBRs while functioning normally (51). Unfortunately, there was no way to find out whether these individuals were present in our sample and, if they were, whether they will only be found in the high-EBR group. In any case, our results suggest that the degree to which a state manipulation affects cognition depends on the individual dopamine level, a trait characteristic (30). This provides an explanation for why the current results in the field of cognitive-enhancement research are so diffuse, and hard to interpret (2). A new neurocognitive approach to cognitive-enhancement techniques should take into account characteristics that can vary between individuals, and which may play a role in how a certain enhancement technique is processed by the brain. Depending on the performance to be enhanced and the characteristics of the task, other individual differences might come into play – neural connectivity, attentional resources, and control styles are likely candidates in this respect. Identifying such candidates requires a basic understanding of the underlying functional and neural mechanisms, which suggests that successful cognitive enhancement is unlikely to work without a guiding theoretical framework.

## Conflict of Interest Statement

The authors declare that the research was conducted in the absence of any commercial or financial relationships that could be construed as a potential conflict of interest.

## Footnotes

^1^Given that Colzato et al. (52) reported higher ABs for low blinkers, we also tested for this effect by calculating the maximal AB in the Control condition for each participant by using Colzato et al.’s formula (T2|T1 at Lag 8 minus the minimum of T2|T1 at Lag3 and at Lag 5) and compared it across the two EBR groups. Numerically, the AB was indeed larger for low blinkers (0.13) than for high blinkers (0.08), but this effect was far from significant, *t*(22) < 1, suggesting that more power might be needed to obtain a stable effect of that sort.
